# An intronic rare mutation in Presenilin-1 (PSEN-1) gene may be involved in the developement of Alzheimer’s disease

**DOI:** 10.1186/1755-8166-7-S1-P25

**Published:** 2014-01-21

**Authors:** Pranami Bhaumik, Priyanka Ghosh, Subrata K Dey

**Affiliations:** 1 Human Genetics Research Laboratory, Department of Biotechnology and Biological Sciences, West Bengal University of Technology, Kolkata 700064, India

## Background

The Presenilin-1 gene (PSEN-1) encodes a protein component of gamma-secretase complex which is involved in processing of amyloid precursor protein (APP). The PSEN-1 is involved in many cardinal mechanisms in several molecular pathway which when impaired leads to the manifestation of Alzheimer’s disease (AD). The aim of the study was to investigate the role of PSEN-1 gene in the developement of AD in Indian Bengali population.

## Materials and methods

Blood samples were collected from 96 AD patients and 173 age matched control individuals. DNA was isolated from each sample and then sequencing was performed for the exon 8 and its flanking introns of PSEN-1 gene.

## Results

A rare mutation rs201992645 was identified within intron 8 and several in. silico analyses (Bioinformatic tools like ‘Human Splicing Finder’, ‘SpliceAid’ and ‘mutation t@sting’) revealed the mutation as ‘potentially damaging’ at the transcript splicing level. The genotypic frequencies of mutant heterozygotes were 0.031 AD, but it was not found in the control population.

## Conclusions

We hypothesize that this rare mutation may be involved in the malfunctioning of Presenilin-1 protein and thus may play a role in the manifestation of Alzheimer’s disease. Further study with large population size may establish this mutation as a potential biomarker for diagnosis of AD.

